# Prodigiosin Demonstrates Promising Antiviral Activity Against Dengue Virus and Zika Virus in In‐silico Study

**DOI:** 10.1002/ansa.202400039

**Published:** 2024-11-07

**Authors:** Tanjilur Rahman, Mohammed Sajjad Hossain Bappi, Tanim Jabid Hossain

**Affiliations:** ^1^ Department of Biochemistry and Molecular Biology University of Chittagong Chattogram Bangladesh; ^2^ Biotechnology, Informatics and Genomics (BIG) Unit, Laboratory for Health Omics and Pathway Exploration (HOPE Lab) Chattogram Bangladesh

**Keywords:** anti‐viral drug | Dengue virus | NS5 methyltransferase | prodigiosin | Zika virus

## Abstract

Dengue (DENV) and Zika virus (ZIKV), transmitted by Aedes mosquitoes, pose significant public health challenges. Effective treatments for these viruses remain elusive, highlighting the urgent need for new efficient antiviral therapies. This study explores prodigiosin, a microbial tripyrrole pigment, as an antiviral agent against both DENV and ZIKV employing advanced analytical approaches which integrate molecular docking, CASTp 3.0 validation and molecular dynamics (MD) simulations providing insights into molecular interactions at an atomic level. Prodigiosin exhibited favourable drug‐likeness properties, meeting Lipinski's rule of five and demonstrating optimal physicochemical and pharmacokinetic characteristics according to Ghose's, Veber's, Egan's and Muegge's filters, essential for oral bioavailability. Absorption, Distribution, Metabolism, Excretion, and Toxicity profiling indicated high intestinal absorption, minimal risk for drug‐drug interactions and a low toxicity profile, with no AMES toxicity, hepatotoxicity, or skin sensitization. Molecular docking revealed prodigiosin's strong binding affinities to NS5 methyltransferases of both DENV (−7.6 kcal/mol) and ZIKV (−7.7 kcal/mol) viruses, suggesting potential disruption of viral replication. Notably, prodigiosin's binding affinities were comparable to ribavirin‐5'‐triphosphate and chloroquine, known inhibitors of DENV and ZIKV, respectively. MD simulations confirmed stable and specific interactions with prodigiosin with low root‐mean‐square deviation values. Additional analyses, including root‐mean‐square fluctuation, radius of gyration and solvent‐accessible surface area, indicated compact and stable complexes. These multi‐parametric in‐silico analytical strategies provide a novel perspective of prodigiosin as an antiviral agent, demonstrating its drug interactions at the molecular level. These promising results suggest that prodigiosin could serve as a broad‐spectrum antiviral agent against both DENV and ZIKV, warranting further experimental validation for therapeutic development against flaviviral infections.

## 1 Introduction

Dengue virus (DENV) and Zika virus (ZIKV) belong to the Flaviviridae family and are transmitted by Aedes mosquitoes. These viruses pose significant risks to public health on a global scale due to their widespread distribution and serious health impacts [1]. DENV is found in over 100 countries and affects approximately 390 million people annually, with about 96 million cases manifesting clinical symptoms [2]. Severe consequences such as dengue hemorrhagic fever and dengue shock syndrome can lead to substantial illness and mortality, especially in tropical and subtropical regions. The ZIKV outbreak in the USA in 2015–2016 also raised global concerns. While ZIKV typically causes mild illness, it is linked with serious birth defects, including microcephaly and neurological conditions such as Guillain‐Barré syndrome [3]. Factors like temperature, humidity and precipitation influence the population of Aedes mosquitoes leading to increased cases and outbreaks of the diseases in regions including the USA, Southeast Asia and the Pacific Islands. Despite the gravity of these diseases, no effective antiviral therapies are currently available [4]. Emphasizing the urgent need for novel therapeutic options [5].

Both DENV and ZIKV share a similar protein structure, consisting of three structural proteins (Capsid [C], Membrane [M] and Envelope [E]) and seven non‐structural proteins (NS1, NS2A, NS2B, NS3, NS4A, NS4B and NS5). Among these, NS5 is notable for its dual functions as an RNA‐dependent RNA polymerase (RdRp) and a methyltransferase (MTase) [6]. The NS5 MTase domain methylates the viral RNA cap process vital for flavivirus replication, making it an attractive target for antiviral drug development. Key areas of the NS5 MTase crystal structure include the SAM‐binding pocket, cap‐binding site and positive RNA‐binding site. Inhibitors like sinefungin and S‐adenosylhomocysteine have targeted these areas but face challenges due to low cellular permeability and lack of selectivity [7]. Similarly, the NS2B/NS3 protease complex is essential for cleaving viral polyproteins into functional proteins required for viral replication [8]. The NS3 protein also functions as a helicase, unwinding double‐stranded viral RNA intermediates during replication [9], ensuring the RNA template is accessible and properly aligned for the NS5 RdRp to synthesize new viral RNA [10]. Given the structural similarities in NS3 helicase domains across flaviviruses, it represents a promising target for broad‐spectrum antiviral drugs that could be effective against multiple viruses.

However, analytical challenges in antiviral research have historically impeded the development of effective treatments. For instance, promising compounds such as balapiravir for Hepatitis C and NITD008 for ZIKV, despite initial success in early studies, ultimately failed in clinical trials due to issues with efficacy and toxicity [11, 12]. Other approaches, including developing short peptides, also fail to show promising results against flavivirus [13]. These failures underscore the broader bottlenecks in drug development, including limitations of traditional models in accurately simulating human pathophysiology and predicting drug behaviour. Consequently, there is an urgent need for novel antiviral agents coupled with advanced analytical strategies to improve the precision of drug selection before clinical trials. Recent advancements in analytical techniques, particularly in‐silico methods, have provided powerful tools to overcome these challenges. By integrating multi‐omics technologies, such as genomics and metabolomics [14, 15, 16, 17], researchers can now identify promising antimicrobial agents and drug candidates more efficiently from both natural and synthetic sources [18]. Computational methods, including Absorption, Distribution, Metabolism, Excretion, and Toxicity (ADMET) profiling and molecular docking, enable precise predictions of pharmacokinetics, drug‐likeness and molecular stability of the drug candidates, addressing many of the challenges in conventional drug discovery.

This study investigates the inhibitory potential of prodigiosin, a natural red pigment, on key proteins of DENV and ZIKV using molecular docking, CASTp 3.0 validation and molecular dynamics (MD) simulations. Prodigiosin, composed of three connected pyrrole rings, is biosynthesized by certain bacterial strains, including *Serratia* [19]. Recently, a strain of *Serratia nematodiphila* has been identified in chrysanthemum rhizospheric soil [20], producing substantial amounts of prodigiosin. Previous research highlights prodigiosin's potent antimicrobial activity against diverse pathogenic bacteria and fungi, along with its documented induction of apoptosis in cancer cells, suggesting potential anti‐cancer properties. Furthermore, prodigiosin exhibits immunosuppressive and anti‐inflammatory effects, which could be beneficial in treating autoimmune diseases and inflammation [21]. Previous studies have also shown that prodigiosin exhibits antiviral properties against human pathogenic viruses, including enterovirus 71 and herpesvirus [22, 23], highlighting its potential utility in developing broad‐spectrum antiviral therapies.

Given its multifaceted biological activities, we hypothesize that prodigiosin could be effective against both DENV and ZIKV, which inhibit proteins essential for their replication. Using molecular docking and dynamic simulations, we aim to evaluate the binding interactions between prodigiosin and critical viral proteins, such as NS5 MTase, NS2B/NS3 protease and NS3 helicase. These multi‐parametric analytical approaches offer a novel perspective on prodigiosin's potential as an antiviral agent, revealing its drug interactions at the molecular level. By offering high‐precision insights into the molecular mechanisms of drug‐target interactions, these methods enable detailed evaluations of binding affinities and the stability of protein‐ligand complexes. Successful binding of prodigiosin to these proteins could impede viral replication, reduce viral loads and offer a foundation for the development of novel antiviral therapies. This computational approach offers a valuable analytical framework to address key challenges in current antiviral drug development.

## 2 Materials and Methods

### 2.1 Analysis of Drug‐Likeness, and ADMET of the Ligand

**FIGURE 1:**
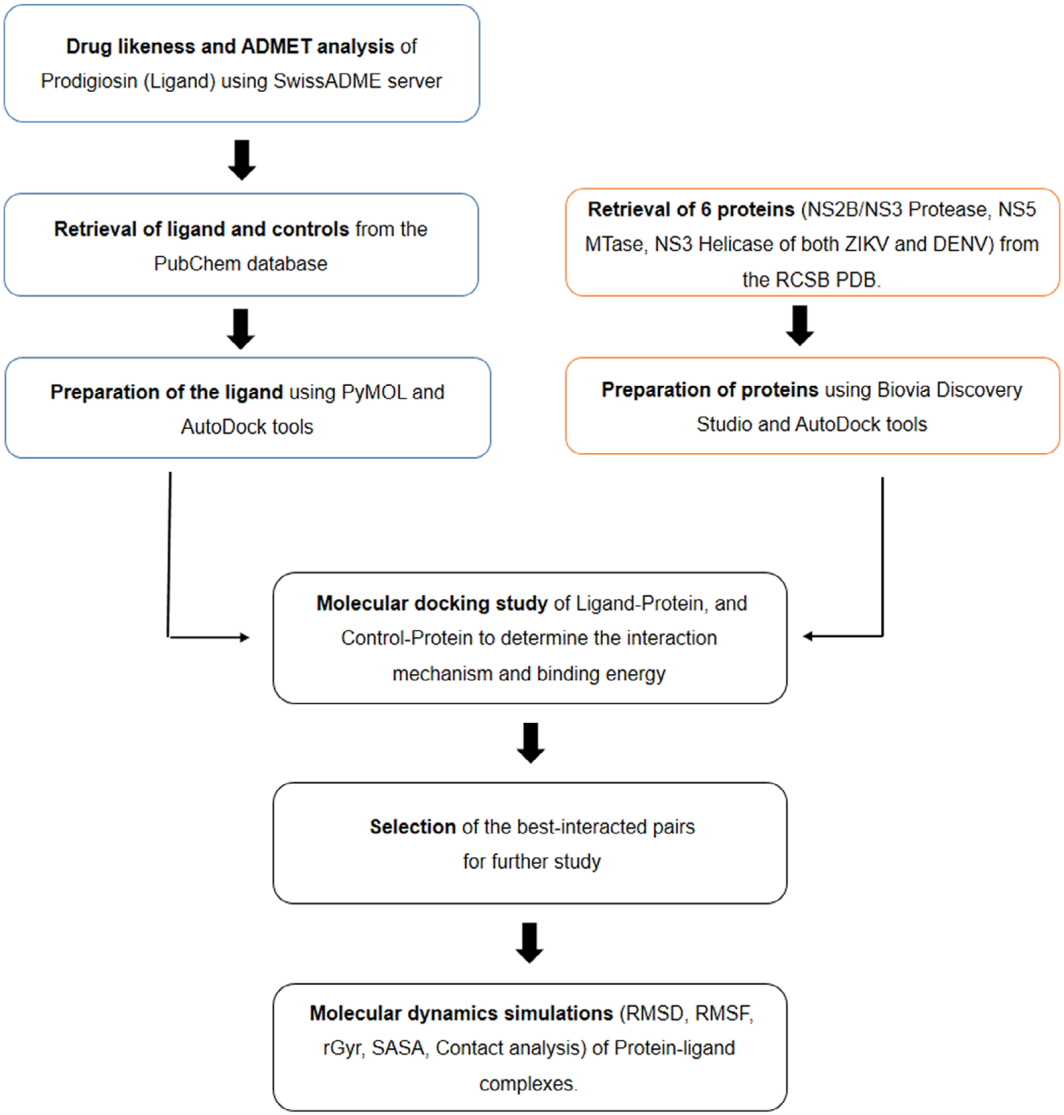
Summary of the methods and tools used in this study. This schematic outlines the workflow of the study, starting with the drug‐likeness and Absorption, Distribution, Metabolism, Excretion, and Toxicity (ADMET) analysis of prodigiosin using the SwissADME server. Ligands and control compounds were retrieved from the PubChem database and prepared using PyMOL and AutoDockTools. Six viral proteins were obtained from the RCSB PDB and processed with Biovia Discovery Studio and AutoDockTools. Molecular docking studies using AutoDock Vina assessed the binding affinities of prodigiosin to viral proteins, identifying the best‐interacted pairs. These pairs were then subjected to molecular dynamics simulations using the Desmond package under the Schrödinger suite, evaluating stability and interactions through parameters like RMSD, RMSF, radius of gyration and solvent‐accessible surface area.

A summary of the computational methods and tools employed in this study has been provided in Figure 1. Prodigiosin, the primary ligand in this study, was subjected to drug‐likeness and ADMET analysis to evaluate its potential as a drug candidate. Drug‐likeness and ADMET properties are vital for determining the viability of a compound in drug development. The SwissADME server (http://www.swissadme.ch/) [24], which predicts drug‐like features and pharmacokinetic properties of small molecules, was used to predict the drug‐likeness of prodigiosin based on several criteria, including Lipinski's five‐criterion rule [25], Ghose's rule [26], Veber's rule [27], Muegge's rule [28], topological polar surface area (TPSA) and the number of rotatable bonds. The SMILES format of prodigiosin was retrieved from the ChEMBL database (https://www.ebi.ac.uk/chembl/) and used as input. For ADMET analysis, the pkCSM server [29] was utilized to analyze the ADMET properties of prodigiosin using the same SMILES format as input.

### 2.2 Bioactivity Analysis of Prodigiosin

The Prediction of Activity Spectra for Substances (PASS) was used to predict the bioactivity spectrum of prodigiosin (http://www.way2drug.com/PASSOnline). PASS predicts over 3500 different types of biological activities, including anticancer, antiulcer, antiviral, antiprotozoal, etc., based on the structure‐activity relationship of over 250,000 compounds [30, 31, 32]. PASS analysis provides two key probabilities: the probability of the molecule being active (Pa) and the probability of it being inactive (Pi). These values range between 0 and 1, with a higher Pa and lower Pi suggesting a greater likelihood that the predicted activity will be experimentally confirmed [33]. In this study, the SMILES format of prodigiosin was submitted to PASS to calculate its predicted bioactivities.

### 2.3 Molecular Docking Study

#### 2.3.1 Ligand Retrieval and Preparation

The 3D structure of prodigiosin was obtained in SDF format from the PubChem database (https://pubchem.ncbi.nlm.nih.gov/) with PubChem CID 135455579. Ribavirin 5’‐triphosphate (PubChem CID: 122108) and Chloroquine (PubChem CID: 2719) were used as control ligands against NS5 MTase of DENV and ZIKV, respectively, and were also retrieved from PubChem in 3D‐SDF format. These SDF files of ligand and controls were converted to PDB format using PyMol v2.5.8 [34] (https://www.pymol.org/) and subsequently to PDBQT format by adding gasteiger charges and merging non‐polar hydrogens using AutoDockTools v1.5.6 [35] (https://autodocksuite.scripps.edu/adt/).

#### 2.3.2 Protein Retrieval and Preparation

Six different proteins were used as receptors for prodigiosin: three from DENV including NS2B/NS3 protease (PDB ID: 2FOM), NS5 methyltransferase (PDB ID: 2P41) and NS3 helicase (PDB ID: 2BMF), and three from ZIKV including NS3 helicase (PDB ID: 5JMT), NS2B/NS3 protease (PDB ID: 5YOD), and NS5 methyltransferase (PDB ID: 5ULP). The 3D structures of these proteins were obtained from the RCSB Protein Data Bank (https://www.rcsb.org/) in PDB format. Preparation of proteins involved removing water molecules and adding polar hydrogen atoms and Kollman charges using Biovia Discovery Studio 2021 [36] (https://www.3ds.com/products/biovia/discovery‐studio) and AutoDockTools v1.5.6. The prepared proteins were saved in PDBQT format for the molecular docking studies. The docking grid box dimensions, set to x: 40, y: 40, z: 40, were determined using AutoDockTools v1.5.6 and remained consistent across all proteins, as detailed in Table 1.

**TABLE 1:** Grid box measurements for all the proteins with grid centre coordinates in the x‐, y‐ and z‐axes.

		DENV			ZIKV		
Center	Axis	NS2B/NS3 Protease	NS3 Helicase	NS5 Methyltransferase	NS2B/NS3 Protease	NS3 Helicase	NS5 Methyltransferase
	X	0.456	−2.641	−13.40	22.740	−12.650	5.269
	Y	−17.036	23.901	77.321	46.048	17.865	1.383
	Z	13.989	39.772	11.674	−21.748	−7.906	27.523

#### 2.3.3 Molecular Docking Investigation

The docking of prodigiosin and control ligands with the viral proteins was performed using Autodock Vina v1.1.2, which estimates binding energy between ligand and receptor molecules using the Lamarckian Genetic Algorithm [37, 38]. Docking was performed with a search space volume of 27,000 Å^3^ and an exhaustiveness of 8 for enhanced results. The software calculates the receptor and ligand's interacting energy and adjusts the complexes’ poses [39]. Input for AutoDock Vina v1.1.2 included PDBQT format files of ligands and proteins. Post‐docking interactions of the best‐docked protein‐ligand complexes were analyzed and visualized using Biovia Discovery Studio 2021. The CASTp 3.0 server was utilized to predict binding pockets for selected proteins [40].

### 2.4 Analysis of MD Simulation

MD simulations were conducted to predict the dynamic properties and structural stability of the protein‐ligand complexes under physiological conditions, evaluating the strength of prodigiosin's binding to the target receptors. Desmond package under the Schrödinger suit was employed to analyze the MD simulation of the best‐docked protein‐ligand complex for 100 ns each [41]. The Protein preparation wizard was used to pre‐process the protein‐ligand complexes prior to simulation [42]. An orthorhombic‐shaped boundary box was assigned for each complex SPC water model with the interval of (10 × 10 × 10 Å^3^). The salt concentration was maintained at 0.15 M by arbitrarily selecting and dispersing Na^+^ and Cl^‐^ ions throughout the solvated system. The OPLS3e force field was applied to reduce and relax the system [43]. Afterwards, 300.0 K temperature and 1.01325 bar pressure were used to complete the constant pressure‐constant temperature (NPT) ensemble [44, 45]. Each complex was first allowed to relax, and then the final analyses were conducted at 100 ps intervals by applying an energy level of 1.2 [46]. After completion of the final production, various dynamics analyses, including root mean square deviation (RMSD), root mean square fluctuation (RMSF), the radius of gyration (rGyr), solvent accessible surface area (SASA) and protein‐ligand interactions, were calculated to assess the stability and flexibility of the complexes.

## 3 Results and Discussion

### 3.1 Drug‐Likeness Properties

To evaluate the potential of prodigiosin as an orally available antiviral candidate, its drug‐likeness was assessed based on multiple pharmacokinetic criteria. Prodigiosin complied with Lipinski's rule of five, which is critical in assessing oral bioavailability. In addition, it met other key criteria, including Ghose's, Veber's and Muegge's filters, indicating that prodigiosin possesses optimal molecular weight, hydrogen bond donors and acceptors, lipophilicity and molar refractivity. It exhibited a bioavailability score of 0.55 and a TPSA of 53.17 Å^2^ (Table 2), supporting its potential for adequate absorption and distribution within the body. These findings indicate that prodigiosin possesses a drug‐like profile, an essential prerequisite for further development as a therapeutic agent.

**TABLE 2:** Drug‐likeness properties of the prodigiosin.

Properties		Prodigiosin (CID 135455579)
**Physico‐chemical properties**	Formula	C_20_H_25_N_30_
	Molecular weight (g/mol)	323.43
	Heavy atoms	24
	Aromatic heavy atoms	10
	Fraction Csp3	0.35
	Rotatable bonds	7
	H‐bond acceptors	2
	H‐bond donors	2
	Molar Refractivity	103.62
	TPSA (Å^2^)	53.17
**Druglikeness**	Lipinski	Yes
	Ghose	Yes
	Veber	Yes
	Egan	Yes
	Muegge	Yes
	Bioavailability Score	0.55

### 3.2 ADMET Profile

In the ADMET analysis, prodigiosin demonstrated a high rate of human intestine absorption and positive Caco‐2 permeability (Table 3). Importantly, it did not function as a substrate or inhibitor of P‐glycoprotein (I and II), suggesting a low potential for drug‐drug interactions. Moreover, the ligand exhibited a moderate blood‐brain barrier (BBB) permeability (Log BB: 0.115) and a low central nervous system (CNS) permeability (Log PS: 2.794), indicating limited brain penetration. Regarding metabolism, prodigiosin was found to be a substrate for CYP3A4 and an inhibitor for CYP1A2 and CYP2C9. The total clearance of prodigiosin was estimated to be 1.048 (log mL/min/kg). Additionally, prodigiosin did not exhibit AMES toxicity, hepatotoxicity, or skin sensitization, indicating a relatively low risk of adverse effects.

**TABLE 3:** Results of Absorption, Distribution, Metabolism, Excretion, and Toxicity (ADMET) analysis of the prodigiosin.

Property	Model name	Predicted value	Property	Model name	Predicted value
**Absorption**	Water solubility	−4.887 (log mol/L)	**Metabolism**	CYP2C19 inhibitor	Yes
	Caco2 permeability	1.35 (log papp in 10^−6^ cm/s)		CYP2C9 inhibitor	No
	Intestinal absorption (human)	89.918%		CYP2D6 inhibitor	No
	Skin permeability	−2.665 (log Kp)		CYP3A4 inhibitor	No
	P‐glycoprotein substrate	No	**Excretion**	Total Clearance	1.048 (log mL/min/kg)
	P‐glycoprotein I inhibitor	No		Renal OCT2 substrate	No
	P‐glycoprotein II inhibitor	No	**Toxicity**	AMES Toxicity	No
**Distribution**	VDss (human)	0.691 (Log L/kg)		Max. tolerated dose (Human)	−0.095 (log mg/kg/day)
	Fraction unbound (Human)	0.243 (Fu)		hERG I inhibitor	No
	BBB permeability	0.115 (log BB)		hERG II inhibitor	No
	CNS permeability	−2.794 (log PS)		Oral Rat Acute Toxicity (LD50)	2.905 (mol/kg)
**Metabolism**	CYP2D6 substrate	No		Oral Rat Chronic Toxicity (LOAEL)	0.47 (log mg/kg_bw/day)
	CYP3A4 substrate	Yes		Hepatoxicity	No
	CYP1A2 inhibitor	Yes		Skin Sensitisation	No

The ADMET profile provided further insights into prodigiosin's pharmacokinetic properties [47]. High human intestinal absorption and positive Caco‐2 permeability indicate that prodigiosin could be efficiently absorbed through the gastrointestinal tract, making it suitable for oral delivery. The absence of P‐glycoprotein interaction reduces the risk of drug‐drug interactions, an important factor since such interactions could compromise the efficacy or safety of co‐administered drugs. Furthermore, prodigiosin's moderate BBB permeability and low CNS permeability indicate limited brain penetration, reducing the risk of adverse effects on the CNS—a critical consideration for antiviral therapies. Additionally, prodigiosin appeared to function as a substrate for CYP3A4 while inhibiting CYP1A2 and CYP2C9 enzymes, implying that it would undergo metabolism by common liver enzymes, while its inhibitory effects could potentially lead to drug interactions or side effects [48]. The absence of AMES toxicity, hepatotoxicity, or skin sensitization indicates a low risk of adverse effects of prodigiosin.

### 3.3 Bioactivity Analysis

PASS analysis revealed that prodigiosin exhibited potential for a broad range of biological activities, with higher Pa and lower Pi values indicating promising activity (Table 4) [49]. Specifically, PASS predicted that prodigiosin had 0.12 to 0.25 for antiviral activities, suggesting a reasonable likelihood of prodigiosin exhibiting antiviral properties. Additionally, prodigiosin demonstrated notable scores for other bioactivities, including Pa values between 0.15 and 0.55 for anticancer activities against several cancers, such as non‐small cell lung cancer, small cell lung cancer, lymphoma and other solid tumours. It also showed Pa values between 0.26 and 0.52 for antiulcer activities, 0.16 to 0.35 for antiprotozoal activities and 0.23 for antibacterial activity against *Helicobacter pylori*. These results suggest that prodigiosin possesses broad‐spectrum bioactivity, enhancing its potential as a therapeutic molecule across multiple diseases.

**TABLE 4:** The bioactivities of prodigiosin.

—	Activity	Pa	Pi
Anticancer	Antineoplastic (non‐small cell lung cancer)	0.549	0.005
	Antineoplastic (solid tumors)	0.428	0.028
	Antineoplastic (small cell lung cancer)	0.342	0.015
	Bcl2 antagonist	0.310	0.003
	Antineoplastic (lymphoma)	0.228	0.040
	Antineoplastic (lymphoma)	0.228	0.040
	Antineoplastic (glioma)	0.211	0.024
	Antineoplastic (thyroid cancer)	0,173	0,068
Antiulcer	Gastrin inhibitor	0.511	0.062
	General pump inhibitor	0.369	0.175
	Antiulcerative	0.277	0.119
Antiulcer	Antiviral	0.216	0.083
	Antiviral (HIV)	0.123	0.09
Antiprotozoal	Antiprotozoal (Amoeba)	0.335	0.047
	Antiprotozoal (Leishmania)	0.219	0.173
	Antiprotozoal (Plasmodium)	0,170	0,090
Antibacterial	Anti‐Helicobacter pylori	0.232	0.080

### 3.4 Molecular Docking

**FIGURE 2:**
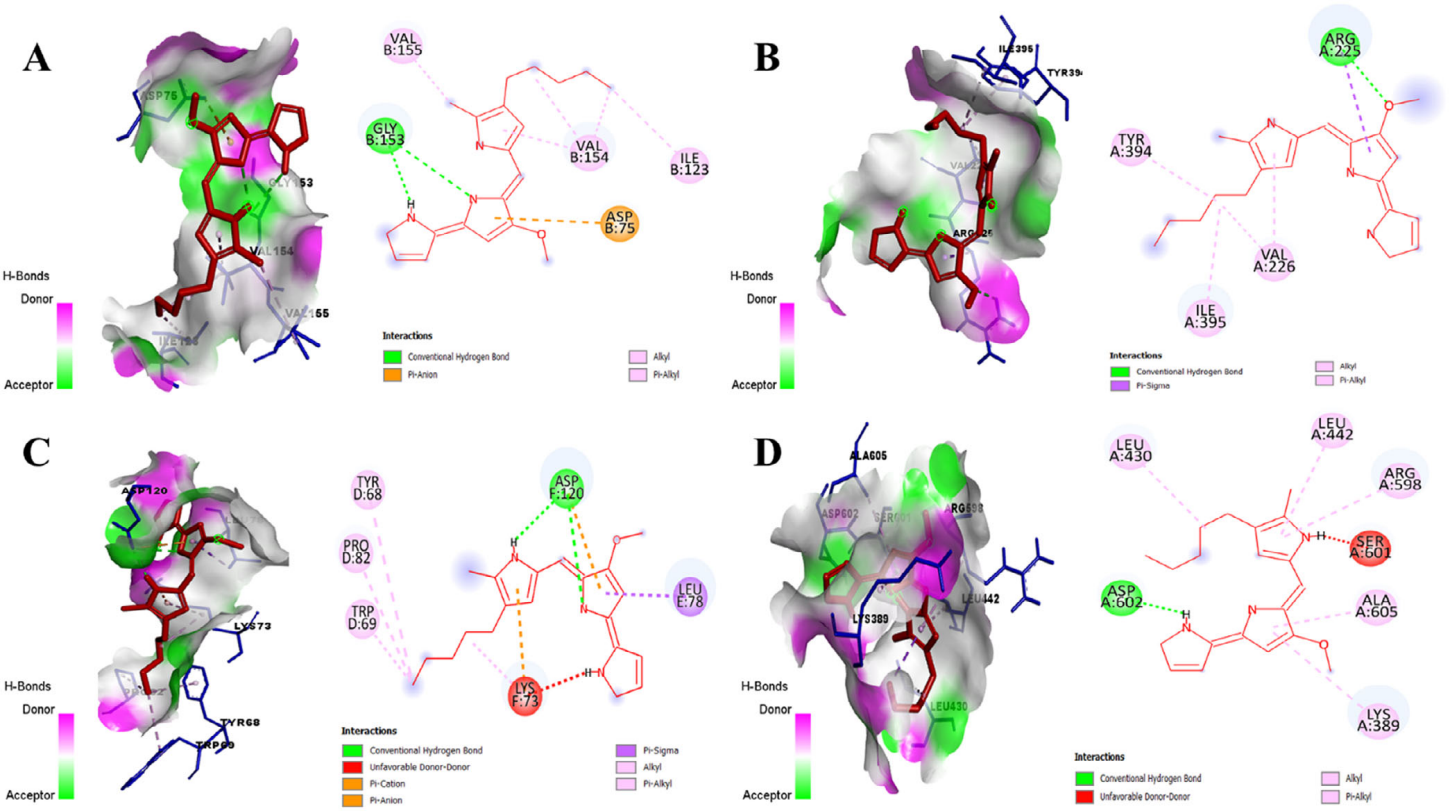
Three‐dimensional (3D) and two‐dimensional (2D) interactions between prodigiosin and viral proteins: (A) Dengue virus (DENV) NS2B/NS3 protease, (B) DENV NS3 helicase, (C) Zika virus (ZIKV) NS2B/NS3 protease and (D) ZIKV NS3 helicase.

**FIGURE 3:**
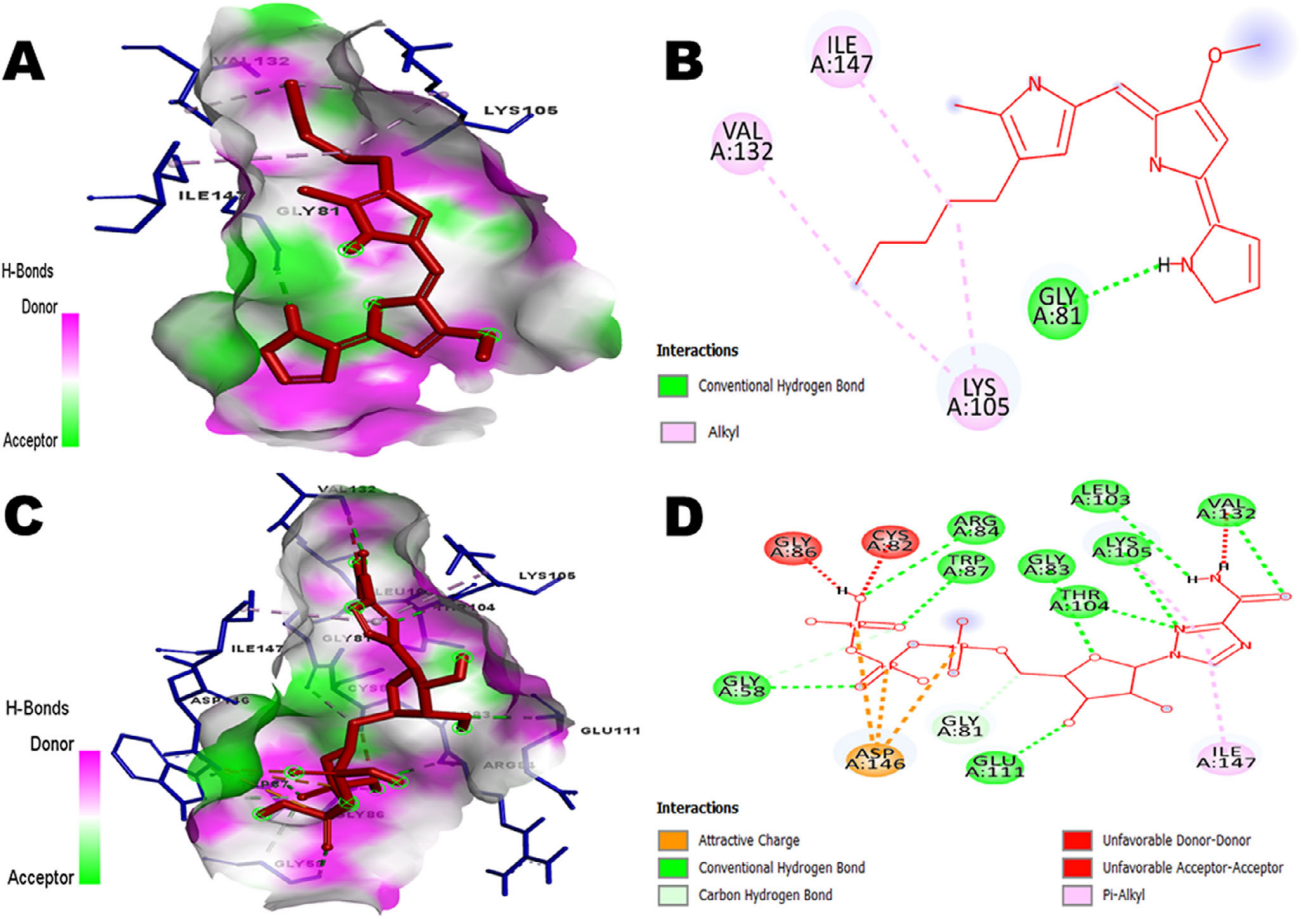
Interactions between Dengue virus (DENV) NS5 MTase and ligands: (A) Three‐dimensional (3D) representation of bonds between DENV NS5 MTase and prodigiosin, (B) Two‐dimensional (2D) representation of bonds between DENV NS5 MTase and prodigiosin, (C) 3D representation of bonds between DENV NS5 MTase and Ribavirin 5’‐triphosphate (control) and (D) 2D representation of bonds between DENV NS5 MTase and Ribavirin 5’‐triphosphate (control).

**FIGURE 4:**
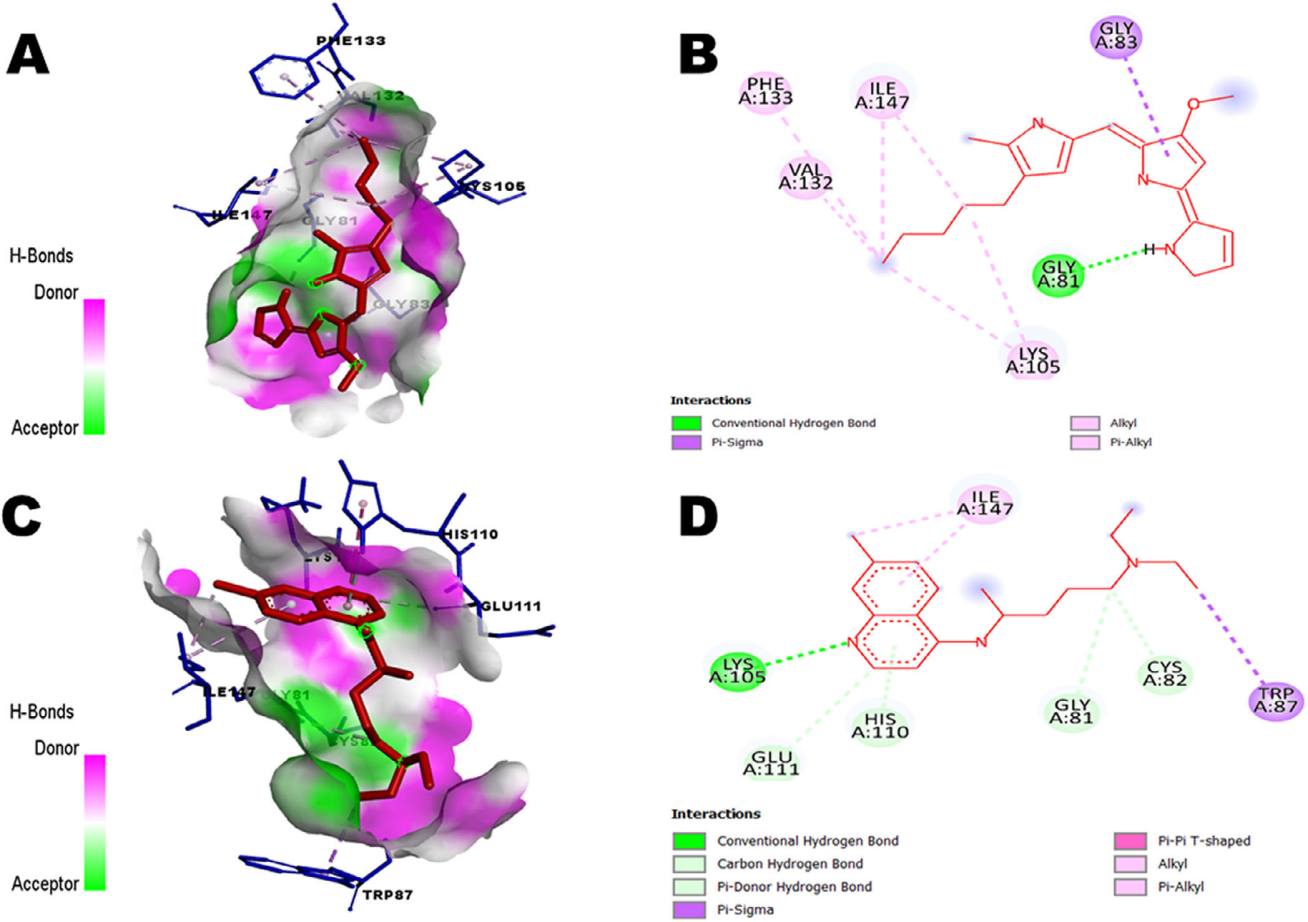
Interactions between Zika virus (ZIKV) NS5 MTase and ligands: (A) Three‐dimensional (3D) representation of bonds between ZIKV NS5 MTase and prodigiosin, (B) Two‐dimensional (2D) representation of bonds between ZIKV NS5 MTase and prodigiosin, (C) 3D representation of interactions between ZIKV NS5 MTase and the control ligand chloroquine (CID: 2719) and (D) 2D representation of interactions between ZIKV NS5 MTase and chloroquine.

Prodigiosin was docked against three viral proteins of DENV (NS2B/NS3 Protease, NS3 helicase and NS5 methyltransferase) and three viral proteins of ZIKV (NS2B/NS3 protease, NS3 helicase and NS5 MTase) to identify the best matches as inhibitors based on binding affinity. Since the lowest binding energy predicts the strongest binding affinity between a ligand and a target receptor [50], the protein with the lowest binding energy for each virus was selected for further analysis. Among the DENV proteins, prodigiosin exhibited binding energies of −7.2 kcal/mol (NS2B/NS3 protease), −6.3 kcal/mol (NS3 helicase) and −7.6 kcal/mol (NS5 MTase). For ZIKV, binding energies were −6.7 kcal/mol (NS2B/NS3 protease), −7.4 kcal/mol (NS3 helicase) and −7.7 kcal/mol (NS5 MTase) (Table 4). Notably, prodigiosin exhibited the strongest binding affinity against NS5 MTases of both DENV and ZIKV. To compare the results, Ribavirin 5’‐ triphosphate, a known inhibitor of DENV NS5 MTase [51], was docked as a positive control. The binding energy of Ribavirin 5’‐triphosphate with DENV NS5 MTase was −7.9 kcal/mol. Additionally, chloroquine, a significant inhibitor of ZIKV NS5 MTase [52, 53], exhibited a binding energy of −6.5 kcal/mol. The detailed molecular docking results are presented in Table 5, and the interactions of all protein‐ligand complexes are depicted in Figures 2, 3, 4. The interactions between prodigiosin and key viral proteins of DENV and ZIKV, as illustrated in Figure 2, reveal significant hydrogen bonding with amino acids of NS2B/NS3 protease and NS3 helicase (GLY153, ARG225, ASP120 and ASP602), along with other bonds including pi‐alkyl, alkyl, pi‐anion, pi‐cation, pi‐sigma, etc. Figure 3 demonstrates the bonding of DENV NS5 MTase with prodigiosin through hydrogen and hydrophobic bonds with residues GLY81, LYS105, VAL132 and ILE147. These residues were also noted during the interaction with the control, Ribavirin 5’‐triphosphate. Similarly, Figure 4 illustrates ZIKV NS5 MTase and prodigiosin interactions compared to the control ligand chloroquine. GLY81 of ZIKV NS5 MTase interacted with prodigiosin via hydrogen bonds, while GLY83 interacted through pi‐sigma bonds. Hydrophobic bonds involving PHE133, VAL132, ILE147 and LYS105 were also observed. Additionally, CASTp 3.0 was used to validate the binding of the ligand in the active site of NS5 MTases of both viruses, with the amino acids present in the binding site listed in Table 6.

**TABLE 5:** Results of molecular dockings and interactions between proteins and ligands.

Ligand	Protein	Organism	Binding Affinity (kcal/mol)	Amino acids	Bonds
Prodigiosin (CID: 135455579)	NS2B/NS3 Protease	DENV	−7.2	ASP75, ILE123, GLY3, VAL154, VAL155	Pi‐Anion, Alkyl, Pi‐Alkyl Hydrogen bond
	NS3 Helicase		−6.3	ARG225, VAL226, TYR394, ILE395	Hydrogen bond, Pi‐Sigma, Alkyl, Pi‐Alkyl
	NS5 Methyltransferase		−7.6	GLY81, LYS105, VAL132, ILE147	Hydrogen bond, Alkyl
	NS2B/NS3 Protease	ZIKV	−6.7	TYR68, TRP69, LYS73, LEU78, PRO82, ASP120	Hydrogen bond, Alkyl, Pi‐Alkyl, Pi‐Sigma
	NS3 Helicase		−7.4	LYS389, LEU430, LEU442, ARG598, ASP602, ALA605	Hydrogen bond, Alkyl, Pi‐Alkyl
	NS5 Methyltransferase		−7.7	GLY81, GLY83, LYS105, VAL132, PHE133, ILE147	Hydrogen bond, Alkyl, Pi‐Alkyl, Pi‐Sigma
Ribavirin 5’‐ triphosphate (CID: 122108)	NS5 Methyltransferase	DENV	−7.9	GLY58, GLY83, ARG84, TRP87, LEU103, THR104, LYS105, GLU111, VAL132	Hydrogen bond, Pi‐Alkyl
Chloroquine (CID: 2719)	NS5 Methyltransferase	ZIKV	−6.5	GLY81, CYS82, LYS105, HIS110, GLU111, ILE147	Hydrogen bond, Alkyl, Pi‐Alkyl, Pi‐Sigma

**TABLE 6:** List of amino acid residues of the predicted active site of NS5 MTase of both Dengue virus (DENV) and Zika virus (ZIKV).

DENV NS5 MTase (PDB ID: 2P41)			ZIKV NS5 MTase (PDB ID: 5ULP)		
Pocket ID	Amino acid	Sequence no.	Pocket ID	Amino Acid	Sequence no.
1	TRP	13	1	LYS	28
1	LYS	14	1	ILE	32
1	LYS	29	1	THR	33
1	GLN	34	1	GLU	34
1	GLU	35	1	VAL	35
1	VAL	36	1	ARG	37
1	ARG	38	1	ARG	41
1	LYS	42	1	SER	56
1	SER	56	1	ARG	57
1	ARG	57	1	GLY	58
1	GLY	58	1	ALA	60
1	LYS	61	1	LYS	61
1	ASP	79	1	ASP	79
1	GLY	81	1	GLY	81
1	CYS	82	1	CYS	82
1	GLY	83	1	GLY	83
1	ARG	84	1	ARG	84
1	GLY	86	1	GLY	85
1	TRP	87	1	GLY	86
1	THR	104	1	TRP	87
1	LYS	105	1	THR	104
1	GLY	106	1	LYS	105
1	GLY	109	1	GLY	106
1	HIS	110	1	GLY	109
1	GLU	111	1	HIS	110
1	VAL	130	1	GLU	111
1	ASP	131	1	VAL	130
1	VAL	132	1	ASP	131
1	PHE	133	1	VAL	132
1	ASP	146	1	ASP	146
1	ILE	147	1	ILE	147
1	GLY	148	1	GLY	148
1	GLU	149	1	GLU	149
1	SER	150	1	SER	150
1	GLU	157	1	LYS	182
1	ARG	160	1	LEU	184
1	ARG	163	1	LEU	211
1	VAL	163	1	SER	212
1	LYS	181	1	ARG	213
1	ASN	184	1	SER	215
1	TYR	186	1	THR	216
1	LEU	210	1	GLU	218
1	SER	211	1	TYR	220
1	ARG	212			
1	SER	214			
1	THR	215			
1	HIS	216			
1	GLU	217			
1	TYR	219			

The docking results confirmed prodigiosin's strong binding affinity for multiple viral proteins, underscoring its potential as a broad‐spectrum antiviral by targeting key pathways through diverse interactions with critical proteins in both DENV and ZIKV. Its binding energies with the NS5 MTases of DENV and ZIKV were particularly notable, showing comparable or even superior binding to established inhibitors such as Ribavirin 5’‐triphosphate and chloroquine. This suggests that the NS5 MTases are particularly promising targets for therapeutic intervention. Given the enzyme's role in viral RNA capping, essential for replication, prodigiosin's inhibition of NS5 MTases could disrupt viral proliferation, reinforcing its potential as a broad‐spectrum antiviral. The reliability of these results was ensured by CASTp 3.0, a powerful tool for identifying, defining and quantifying the topological and geometric characteristics of protein structures. The analysis confirmed that prodigiosin would accurately bind within the NS5 MTase active site. These findings can guide future efforts in structure‐based drug design and optimization.

### 3.5 MD Simulation

MD simulations were performed to investigate the stability and dynamics of the protein‐ligand complexes between prodigiosin and the target proteins under specific physiological conditions. Since prodigiosin demonstrated the highest binding affinity against DENV NS5 MTase and ZIKV NS5 MTase in molecular docking analysis, these proteins with their ligand prodigiosin (CID: 135455579) were selected for MD simulation. Additionally, the complexes of the control ligands Ribavirin 5’‐triphosphate (CID: 122108) and Chloroquine (CID: 2719) of DENV NS5 MTase and ZIKV NS5 MTase, respectively, were also subjected to MD simulations for comparative analysis. Various parameters, including RMSD, RMSF, SASA, rGyr and protein‐ligand interactions, were analyzed to assess the stability and conformational changes in the proteins upon ligand binding, further reinforcing the molecular docking results.

#### 3.5.1 Root Mean Square Deviation

**FIGURE 5:**
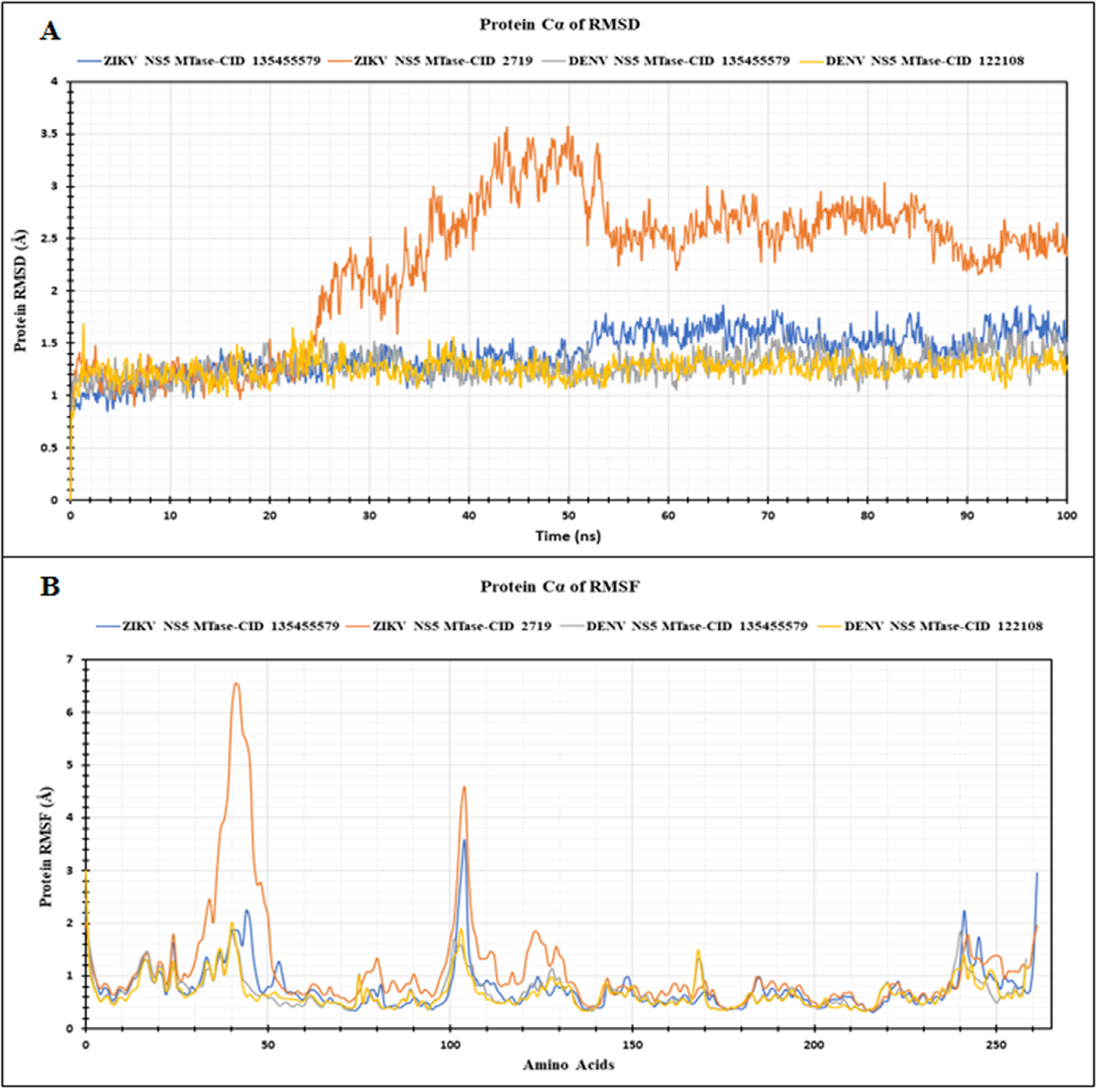
Root Mean Square Deviation (RMSD) and Root Mean Square Fluctuation (RMSF) analyses of NS5 MTase proteins: (A) RMSD values for the NS5 MTase proteins in four complexes: Zika virus (ZIKV) NS5 MTase with prodigiosin (CID 135455579) (blue), ZIKV NS5 MTase with chloroquine (CID 2719) (orange), DENV NS5 MTase with prodigiosin (CID 135455579) (ash) and Dengue virus (DENV) NS5 MTase with ribavirin 5’‐triphosphate (CID 122108) (yellow). (B) RMSF values for the same protein‐ligand complexes, with the colour scheme corresponding to the same complexes as in (A).

RMSD measures the deviation of a protein's backbone structure in a protein‐ligand complex relative to its initial conformation, providing insights into the protein's stability, dynamic properties and conformational changes during the simulation [54, 55]. Figure 5A presents the RMSD analysis of the four protein‐ligand complexes: DENV NS5 MTase‐prodigiosin (CID 135455579), DENV NS5 MTase‐ribavirin 5’‐ triphosphate (CID 122108), ZIKV NS5 MTase‐prodigiosin (CID 135455579) and ZIKV NS5 MTase‐chloroquine (CID 2719). Both DENV and ZIKV NS5 MTases demonstrated remarkable stability when bound to prodigiosin. The low RMSD values of 1.28 Å for the DENV NS5 MTase‐prodigiosin complex and 1.41 Å for the ZIKV NS5 MTase‐prodigiosin complex indicate minimal structural deviations and strong interactions during the simulation. In contrast, chloroquine (CID 2719), a positive control for the ZIKV NS5 MTase, exhibited a higher RMSD of 2.26 Å when bound to ZIKV NS5 MTase, indicating relatively less stability and greater structural fluctuations compared to prodigiosin. Interestingly, the DENV NS5 MTase‐Ribavirin 5’‐triphosphate (CID 122108) complex showed an average RMSD of 1.26 Å, similar to the DENV NS5 MTase‐prodigiosin complex, suggesting comparable stability and effective binding. Among the four complexes, the highest RMSD values recorded were 1.66, 1.69, 1.87 and 3.57 Å, respectively, and the lowest RMSD values were 0.96, 0.79, 0.85 and 0.89 Å, respectively.

The low RMSD values observed for prodigiosin in both DENV and ZIKV NS5 MTase complexes indicate strong structural stability, suggesting that prodigiosin maintained stable interactions with the target proteins over the simulation period. In comparison, the higher RMSD for chloroquine suggests that prodigiosin might confer greater stability to the ZIKV NS5 MTase complex. This stability is vital for the effectiveness of antiviral drugs, as maintaining consistent binding with viral proteins can disrupt their function, thereby inhibiting viral replication.

#### 3.5.2 Root Mean Square Fluctuation

RMSF evaluates local fluctuations in the protein chain when ligands interact with its specific residues. In this study, RMSF analysis was conducted on the NS5 MTase proteins of DENV and ZIKV to understand their structural flexibility upon binding with prodigiosin (CID 135455579), or the control ligands Ribavirin 5’‐triphosphate (CID 122108) and chloroquine (CID 2719) for DENV and ZIKV NS5 MTases respectively (Figure 5B). Prodigiosin exhibited distinct effects on both viral proteins. DENV NS5 MTase showed RMSF values of 0.70 Å with prodigiosin and 0.68 Å with Ribavirin 5’‐triphosphate, implying similar structural stability in both complexes. Conversely, for ZIKV NS5 MTase, prodigiosin resulted in an average RMSF value of 0.78 Å, indicating relatively low local fluctuations and stable binding, in contrast to chloroquine, which showed an average RMSF value of 1.14 Å. Furthermore, the analysis revealed that ZIKV NS5 MTase exhibited the highest fluctuations at residues 29–52 and 98–106 when bound to chloroquine while achieving greater stability with prodigiosin. For the DENV NS5 MTase, similar fluctuations were observed regardless of whether the ligand was prodigiosin or Ribavirin 5’‐triphosphate. Both proteins regardless of any ligands, demonstrated stability in the region of residues 140–240 with the highest fluctuations noted at the N‐ and C‐terminal regions of the proteins, likely due to the inherent flexibility of N‐ and C‐terminals domains in the proteins. The RMSF analysis confirmed prodigiosin's stabilizing effect on the NS5 MTase proteins of both viruses. The lower RMSF values observed for prodigiosin compared to chloroquine in ZIKV NS5 MTase, indicate fewer local fluctuations and a more stable interaction.

#### 3.5.3 Radius of Gyration

**FIGURE 6:**
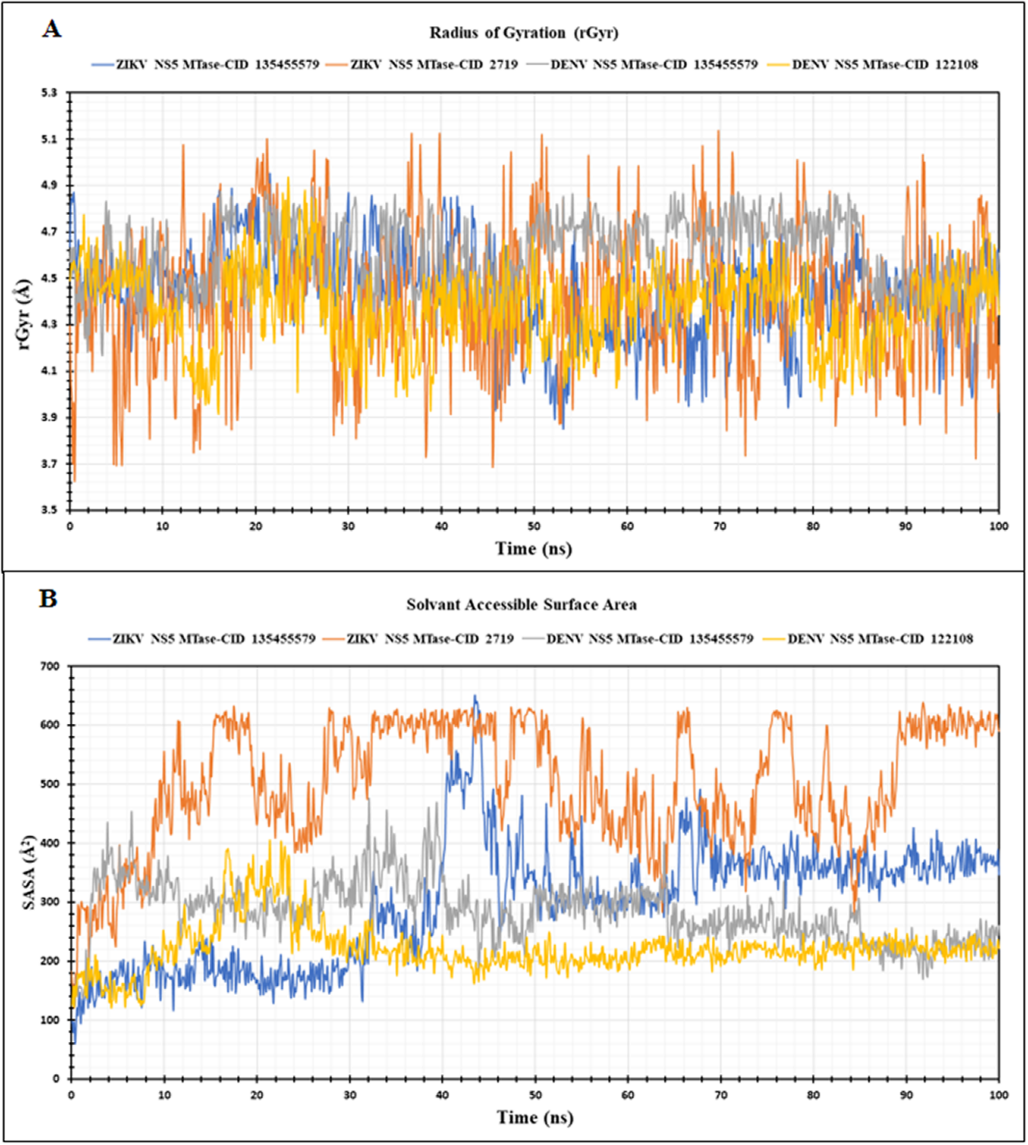
Radius of Gyration (rGyr) and Solvent Accessible Surface Area (SASA) analysis of protein‐ligand complexes: (A) The radius of gyration (rGyr) values for the four complexes are shown, with Zika virus (ZIKV) NS5 MTase—CID 135455579 (prodigiosin) in blue, ZIKV NS5 MTase—CID 2719 (chloroquine, control) in orange, Dengue virus (DENV) NS5 MTase—CID 135455579 (prodigiosin) in ash and DENV NS5 MTase—CID 122108 (ribavirin 5'‐triphosphate, control) in yellow. (B) The Solvent Accessible Surface Area (SASA) for NS5 MTase proteins in the four complexes is depicted, utilizing the same colour coding as in (A) to represent the respective complexes.

The spatial arrangement of atoms within a protein‐ligand complex with respect to its axis is known as the radius of gyration, or rGyr. The rGyr analysis was performed to assess the compactness of the four protein‐ligand complexes in our study (Figure 6A). For the DENV NS5 MTase complex with prodigiosin (CID 135455579), the average rGyr was 4.60 Å, slightly higher than the average rGyr of 4.44 Å observed with the control ligand Ribavirin 5'‐triphosphate (CID 122108). In the case of the ZIKV NS5 MTase—prodigiosin (CID 135455579) complex, the average rGyr value was 4.44 Å, indicating a stable and compact structure. This was comparable to the rGyr value of the ZIKV NS5 MTase—chloroquine (CID 2719) complex, which was 4.43 Å, suggesting that prodigiosin maintains the structural integrity of the ZIKV NS5 MTase complex similar to the known inhibitor chloroquine.

#### 3.5.4 Solvent‐Accessible Surface Area

The SASA measures the area of a protein's surface that can interact with a solvent, which is essential for understanding protein stability, folding and interactions in various solvent environments. SASA values for the four selected complexes are presented in Figure 6B. Prodigiosin (CID 135455579) induced a more compact and less solvent‐exposed structure in both complexes of DENV and ZIKV NS5 MTases, with relatively low average SASA values of 296.84 and 284.76 Å^2^, respectively. However, the ZIKV NS5 MTase‐chloroquine (CID 2719) complex exhibited a significantly higher SASA value of 500.30 Å^2^ compared to the ZIKV NS5 MTase‐prodigiosin (CID 135455579) complex, suggesting that prodigiosin binding resulted in a considerable reduction of solvent‐exposed surface area. In contrast, ribavirin 5'‐triphosphate (CID 122108), the documented inhibitor of DENV NS5 MTase, exhibited a SASA value of 221.68 Å^2^ when bound to DENV NS5 MTase, indicating a similar effect as prodigiosin on the compactness and solvent exposure of DENV NS5 MTase.

These results support the compact and stable nature of the prodigiosin‐bound complexes with lower solvent exposure, suggesting that prodigiosin binding stabilizes the protein structure and potentially reduces its susceptibility to degradation. Besides, the reduction in solvent‐exposed surface area indicates a more stable protein‐ligand complex, favorable for drug efficacy.

#### 3.5.5 Protein‐Ligand Contact Analysis

**FIGURE 7:**
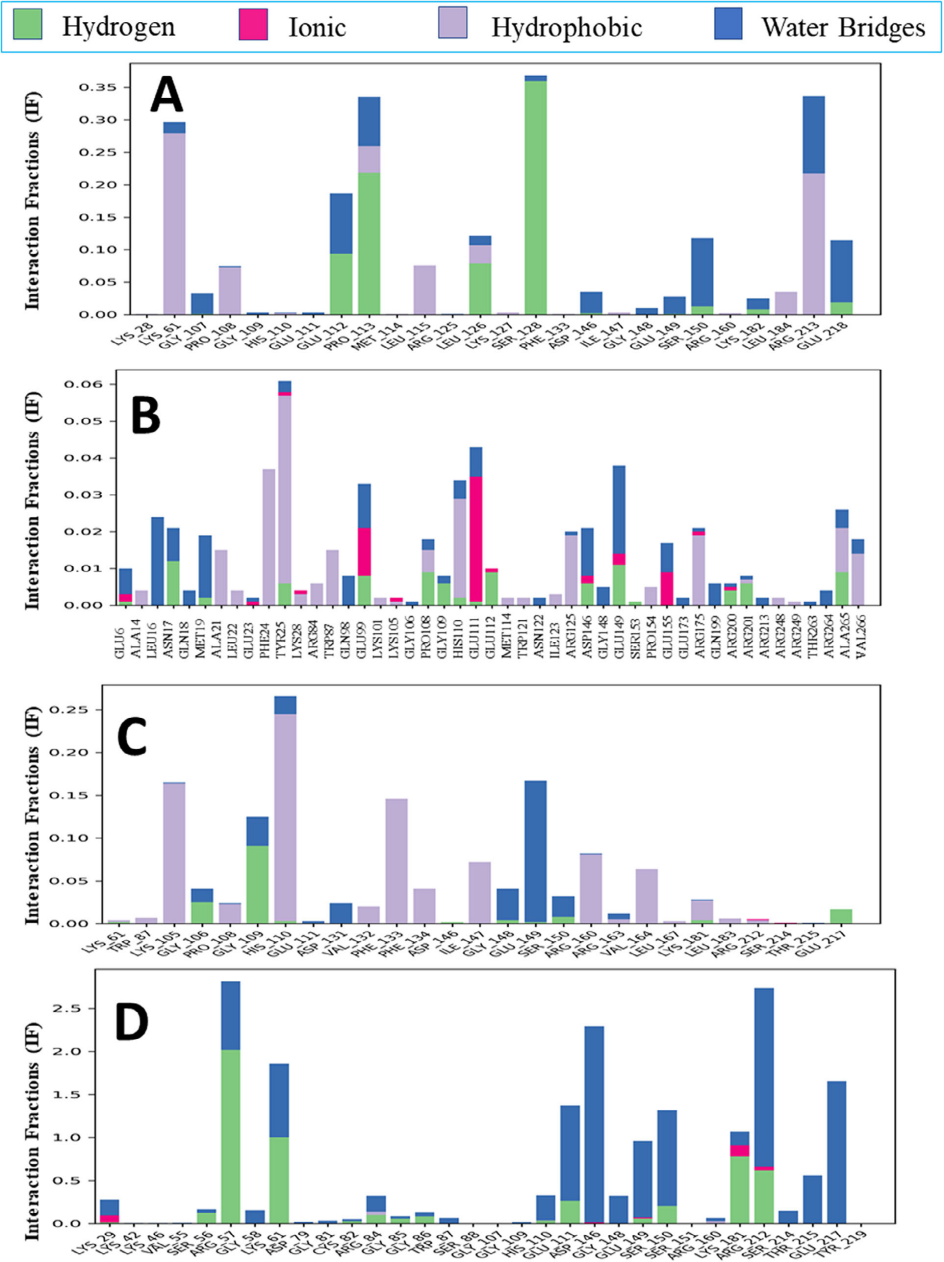
Bar charts presenting the interactions of protein‐ligand complexes during the 100 ns simulation. (A) Interactions between Zika virus (ZIKV) NS5 MTase and prodigiosin (CID 135455579), (B) Interactions between ZIKV NS5 MTase and chloroquine (CID 2719). (C) and (D) Interactions between Dengue virus (DENV) NS5 MTase and prodigiosin (CID 135455579) and DENV NS5 MTase and ribavirin 5'‐triphosphate (CID 122108), respectively.

Protein‐ligand interactions and the architecture of the protein‐ligand complexes were analyzed via the simulation interaction diagram during the 100 ns MD simulation. The interactions between the proteins and ligands in our four complexes are shown in Figure 7. These interactions encompassed various bond types, including ionic bonds, hydrogen bonds, non‐covalent bonds and water bridges. Prodigiosin (CID: 135455579) made interactions with the ZIKV NS5 MTase receptor at PRO113 and LEU126, with interaction fractions (IF) of 0.33 and 0.13 respectively (Figure 7A). In contrast, the control ligand chloroquine (CID: 2719) interacted with ZIKV NS5 MTase at GLU6, TYR25, GLU99, PRO108, HIS110, GLU111, ASP146, GLU149, ARG175, ARG200, ARG201 and ALA265, with IF values ranging from 0.01 to 0.06 (Figure 7B). Prodigiosin (CID: 135455579) also showed interactions with DENV MTase at HIS110 and LYS181, with IF values of 0.25 and 0.03 respectively (Figure 7C). Conversely, Ribavirin 5’‐triphosphate (CID 122108) interacted with the protein at LYS29, ARG84, HIS110, GLU149, LYS181 and ARG212, with IF values ranging from 0.3 to 2.6 (Figure 7D). The protein‐ligand contact analysis revealed multiple stable interactions of prodigiosin with key residues in both DENV and ZIKV NS5 MTases, highlighting the strength and specificity of prodigiosin's binding to critical viral proteins, supporting its potential to inhibit viral replication.

Overall, the MD simulation results, combined with the molecular docking studies, suggest that prodigiosin is a promising candidate for further development as a broad‐spectrum antiviral agent against flaviviruses. Additional in vitro and in vivo tests are needed to confirm prodigiosin's utility against the target proteins in drug design.

## 4 Conclusion

Based on the comprehensive analysis conducted in this study, prodigiosin has demonstrated promising potential as a broad‐spectrum inhibitor of key proteins in DENV and ZIKV. Through computational approaches including molecular docking and MD simulations, prodigiosin exhibited strong binding affinities with NS5 methyltransferases of both viruses, which are essential for viral replication. This interaction was supported by stable protein‐ligand complexes observed in MD simulations, suggesting effective inhibition mechanisms. Furthermore, prodigiosin met drug‐likeness criteria and showed favourable ADMET properties, indicating its potential as a safe and effective therapeutic candidate. These findings underscore the utility of prodigiosin as a promising lead compound for antiviral development against DENV and ZIKV infections. Its broad‐spectrum potential also opens avenues for developing versatile antiviral treatments, addressing the urgent need for effective therapeutics targeting flaviviruses. Given the global burden of flaviviral infections, particularly dengue and Zika, the development of new therapeutic options is of significant importance. The promising in‐silico performance of prodigiosin can be a key step forward in this search for effective treatments against these viruses. However, the reliance on in‐silico analyses necessitates further experimental validation, including in‐vitro and in‐vivo studies, to confirm prodigiosin's efficacy and safety in biological systems. Future research should also investigate its mechanism of action in greater detail and evaluate its potential synergy with existing antiviral drugs. Structural optimization could further enhance prodigiosin's antiviral activity and pharmacological profile, paving the way for novel therapeutic strategies. In this respect, the findings of this study not only highlight prodigiosin's potential but also provide valuable insights for advancing antiviral drug development, offering a pathway for its inclusion in antiviral drug pipelines and contributing to the urgent need for innovative therapies against flaviviruses and related viral pathogens. Overall, prodigiosin represents a promising candidate for further development, contributing to the urgent need for effective treatments against flaviviral infections.

## Author Contributions

Tanim Jabid Hossain and Mohammed Sajjad Hossain Bappi conceived the idea. Tanjilur Rahman and Mohammed Sajjad Hossain Bappi designed and conducted the study. Tanjilur Rahman and Mohammed Sajjad Hossain Bappi wrote the first draft. Tanim Jabid Hossain revised the manuscript.

## Conflicts of Interest

The authors declare no conflicts of interest.

## Data Availability

All data are available within the article.
